# Impact of the COVID-19 pandemic on surgery for severe endometriosis in the UK: a national database study

**DOI:** 10.52054/FVVO.14.4.043

**Published:** 2023-01-27

**Authors:** J Lewin, E Saridogan, D Byrne, T.J. Clark, A Vashisht

**Affiliations:** University College London Hospital, Women’s Health Division, London United Kingdom; Department of Gynaecology, Royal Cornwall Hospital, Truro, United Kingdom; Birmingham Women’s and Children’s Hospital, Birmingham, United Kingdom

**Keywords:** Endometriosis, laparoscopic surgery, COVID-19, coronavirus, Minimal access surgery

## Abstract

**Background:**

The COVID-19 pandemic has had a significant effect on healthcare services, particularly affecting patients who suffer from chronic conditions. However, the pandemic’s effect on endometriosis surgery is not yet known.

**Objectives:**

To determine the impact of the COVID-19 pandemic on surgery for severe endometriosis in the UK at a national, regional and centre-level.

**Materials and Methods:**

The British Society for Gynaecological Endoscopy (BSGE) collects data nationally on all operations for severe endometriosis which involve dissection of the pararectal space. Annual audits of this database were obtained from the BSGE. Publicly available data on COVID-19 cases and population were obtained from the UK Office for National Statistics.

**Main outcome measures:**

Numbers of annual BSGE-registered endometriosis operations.

**Results:**

A total of 8204 operations were performed. The number of operations decreased by 49.4% between 2019 and 2020 and then increased in 2021, but remained 10.5% below average pre-pandemic levels, indicating at least 980 missed operations between 2019-2020. Median operations per centre decreased by 51.0% in 2020 (IQR 29.4% – 75.0%) and increased in 2021 but remained 33% below pre-pandemic levels. There was no change in the type of surgery performed. All 11 administrative regions of Great Britain had reduced numbers of operations in 2020 compared with the average for 2017-2019, with a median 44.2% decrease (range 13.3% - 67.5%). Regional reduction in operations was correlated with COVID-19 infection rates (r=0.54, 95% CI of r 0.022 – 1.00, p=0.043).

**Conclusion:**

The number of operations performed annually in the UK for severe endometriosis fell dramatically during the COVID-19 pandemic and is yet to normalise.

**What's new?:**

This study shows the dramatic effect that the COVID-19 pandemic has had on UK services for endometriosis surgery, which may continue to affect patients and clinicians for a considerable time to come.

## Introduction

The coronavirus pandemic has had a significant impact on surgical services in the UK, with more than 1.5 million operations cancelled or postponed in 2020 alone (Dobbs et al., 2021). To increase ITU capacity to care for ventilated patients, operating theatres throughout the country were transformed into intensive care or high-dependency units. Anaesthetists required for elective surgery were unavailable as their unique skills in caring for the critically ill were required for COVID patients. Most other non-emergency teams and healthcare professionals were redeployed to help as frontline workers to combat the pandemic and treat afflicted patients. Many other staff have been unable to work in operating theatres due to illness or shielding for pre-existing health conditions. Patients have also faced reduced access to primary care services and specialist surgical clinics, further preventing or delaying the scheduling of operations (NHS England, 2021a; Turner et al., 2021). As of December 2021, over 6 million patients in the UK were waiting to start elective care (The Nuffield Trust, 2022), and this unprecedented backlog may take years to address (BMA, 2022).

Patients with endometriosis wait on average 8 years for a diagnosis alone, and 30% wait more than 10 months for surgery (APPG on endometriosis, 2020). The delay in surgery incurs secondary economic costs such as time out of education and employment, and places additional strains on emergency services with attendances for uncontrolled pain. It is vital that the backlog of endometriosis surgery is quantified, and a plan is made to reduce the waiting times for endometriosis surgery to a sustainable level.

The British Society for Gynaecological Endoscopy (BSGE) is an organisation set up by clinicians to improve standards, training, and exchange of information in gynaecological minimal access surgery. It is the primary national aggregator of data on endometriosis surgery in the UK. The purpose of this study is to assess the impact of COVID-19 on surgery for severe endometriosis at a national, regional and centre- level in the UK using the BSGE database, and to assess the correlation between regional pandemic severity and disruption to these services.

## Methods

The BSGE maintains a national accreditation process for centres specialising in the care of patients with severe endometriosis, and a database of operations at these centres. To be granted BSGE annual accreditation as an endometriosis centre, at least 12 cases of laparoscopic surgical excision of rectovaginal endometriosis requiring dissection of the pararectal space per surgeon are required, and any such case must be registered on the national BSGE database. Auditing of case numbers at each centre is performed annually by the BSGE.

Annual audits of the BSGE database were obtained from the BSGE endometriosis centres committee including data from all accredited centres from 2017 – 2021 inclusive to analyse the impact of the COVID-19 pandemic. Data included the number of operations performed annually at each centre and the type of bowel surgery performed. Region- level data on operations were aggregated according to the region of the centre at which the operations were performed. Centres on the database which did not enter any cases at all were also excluded. The audit data is classed as service evaluation data, is publicly available and is fully anonymised, so prior ethical approval was not required (HRA, 2017). Comparison of COVID case rates with operation rates were not performed for Northern Ireland due to differences in COVID reporting.

Data on regional COVID case rates were obtained from publicly available data compiled by the UK Office of National Statistics (ONS) (ONS, 2021a) and the National Registry of Scotland(NRS, 2022). Cases data includes all positive lab-confirmed PCR test results plus positive rapid lateral flow tests, unless followed by a negative PCR test taken within 72 hours. Regional population estimates were obtained from the ONS (ONS, 2021b) and used to calculate registered COVID cases per 1000 population. Statistical analysis was performed using Pearson r correlation coefficient and Chi2 goodness-of-fit test in R with the Base and Tidyverse packages. Graphs were plotted in R using RStudio with the ggplot2 package, and regional mapping templates were obtained from the ONS (ONS, 2018).

## Results

### Centres

**Figure 1:**
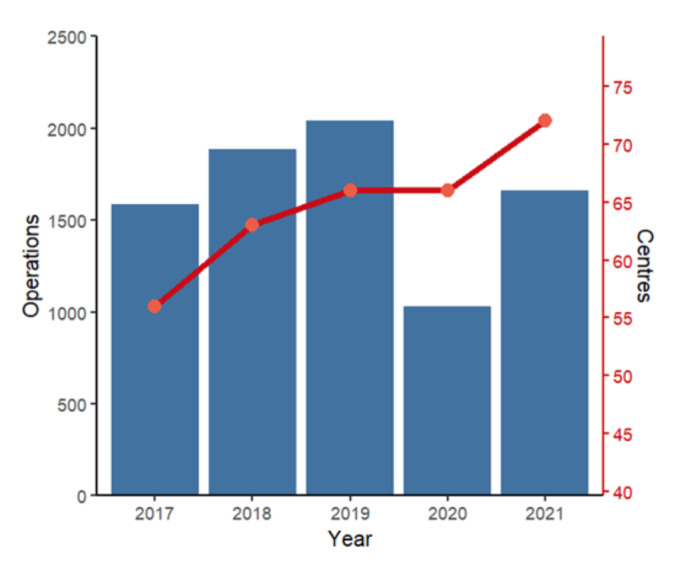
Time schedule and measured variables. EA: endometrial ablation.

A total of 81 centres were registered with the BSGE between 2017 to 2021. Over this period, the number of centres which submitted data for registered operations each year increased from 56 to 72 (Figure 1). Four centres submitted registered operations for the first time in 2020, and 8 centres for the first time in 2021. Out of 81 centres, 45 submitted registered operations every year, 12 for four out of the 5 years, 11 only for 3 years, 4 only for 2 years and 9 only for 1 year.

### Operations

**Figure 2:**
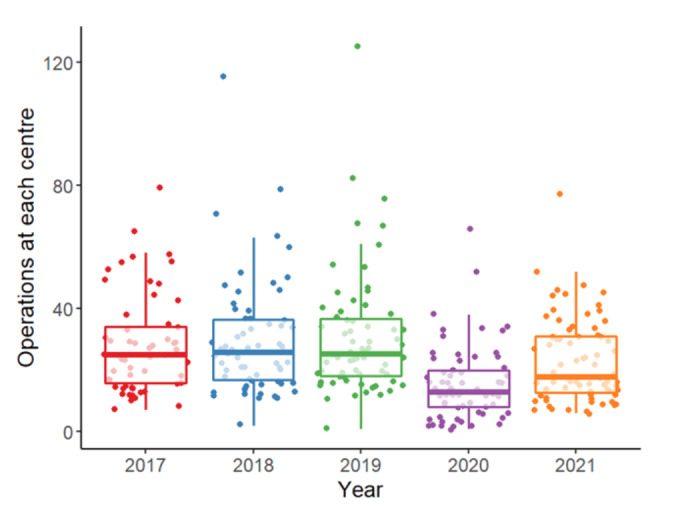
Boxplot of number of BSGE registered operations in each year at each endometriosis centre. Each datapoint represents data from one centre in each year.

Over the period from 2017 – 2021, a total of 8204 operations were performed for deep endometriosis requiring pararectal dissection at BSGE-accredited centres (Figure 2). The total number of registered operations increased by an average of 13.5% per year between 2017-2019, then decreased by 49.4% between 2019-2020, from 2040 operations in 2019 to 1032 operations in 2020. The number of operations increased in 2021 to 1662, remaining 10.5% below the average of 1837 registered operations per year for 2017- 2019. Over 2020 and 2021, there were a total of 980 fewer operations compared to the average for 2017-2019.

At a centre level, 64 endometriosis centres had reduced operation numbers in 2020 compared with the average for 2017-2019, 7 of which were not able to submit any cases in 2020. Five centres had increased numbers of operations in 2020, while 4 centres registered cases for the first time. Overall, the annual median number of operations per centre decreased from 24 operations in 2019 to 12 operations in 2020, with a median decrease in operations per centre of 56.1% (IQR 37.2% – 75.4%). Between 2020 and 2021, 49 centres had increased operations, 15 centres had decreased operations including 9 centres not registering any cases, while 9 centres had no change.

Between 2020-2021, registered operations increased by a median of 48% at each centre (IQR 0.0% – 166.7%). However, 15 centres submitted fewer registered operations in 2021 than 2020, and 9 previously accredited endometriosis centres did not submit any cases to the database in 2021. The number of registered operations at each centre in 2021 remained lower than the 2017-2019 average by a median of 33.3% (IQR 4.2% - 59.2%).

### Type of bowel surgery performed

**Figure 3:**
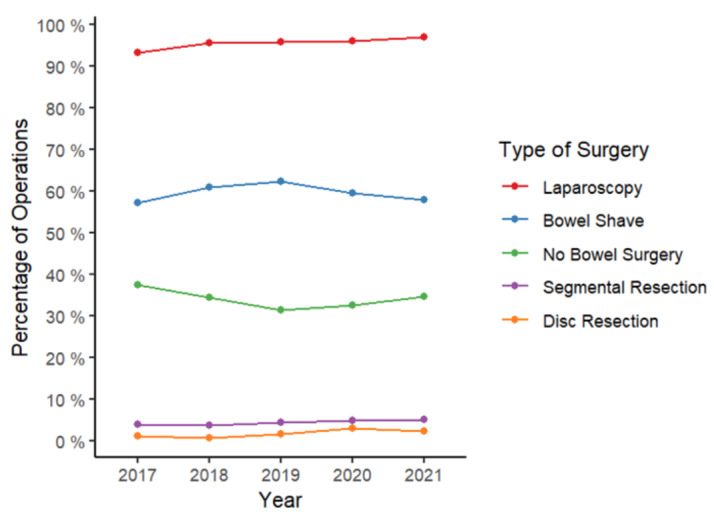
Trend in type of bowel surgery performed.

Between 2017 and 1019, on average 34.4% of patients had no surgery on the bowel, 60.2% underwent shaving of endometriosis from the bowel surface, 1.3% underwent a disc resection and 4.1% underwent resection of a segment of bowel (Figure 3), and there was little change in these proportions in 2020 and 2021. A laparoscopic approach remained high (95% or greater) from 2018 to 2021.

### Regional Impact of COVID

**Figure 4:**
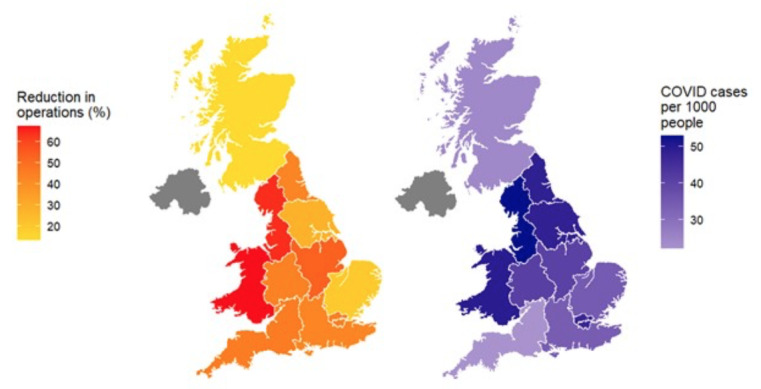
Geographic plot of reduction in operations compared to previous 3-year average (left) and COVID deaths per 1000 population.

At a regional level, all 11 administrative regions of Great Britain had reduced numbers of BSGE- registered operations for endometriosis in 2020 compared with averages for 2017-2019, with a median 44.2% decrease (range 13.3% - 67.5%) (Figure 4). The regions with the greatest reduction in operations were Wales (67.5%) and the Northwest of England (64.6%), while the least- affected region was Scotland (13.3%).

Regions had a median of 40.9 cases of COVID- 19 per 1000 population in 2020 (range 22.0 – 52.7). At a regional level, reduction in BSGE- registered operations was significantly correlated with COVID-19 case rates (r=0.54, 95% CI of r 0.022 – 1.00, p=0.043).

## Discussion

### Main findings

Our study demonstrates the concerning effect of the COVID-19 pandemic on surgery for severe endometriosis in the UK. We found a decline in registered operations by nearly 50% in 2020, which was significantly higher than the 38.6% reduction in elective procedures nationwide (Dobbs et al., 2021). This may indicate that endometriosis surgery has been particularly badly affected, which would be in keeping with evidence that women and patients with chronic conditions suffered significantly higher cancellations of care during lockdown periods (Topriceanu et al., 2021). Although the number of recorded operations increased in 2021, this figure remained below the average for 2017-2019, indicating a significant estimated shortfall of 980 operations between 2020 and 2021.

### Strengths and Limitations

This study is the first to provide a comprehensive assessment of the effect of the COVID-19 pandemic on surgery for severe endometriosis in the UK. A nation-wide, standardised approach was taken in data collection, however only BSGE-registered operations were considered, and there may have been other operations performed which were not registered with the BSGE. There was also a lack of data from Northern Ireland, restricting analysis to Great Britain.

### Interpretation

The magnitude of this reduction in operations for a common, chronic, gynaecological condition, highlights the challenges for restoration of endometriosis services. There is a pressing need in the pandemic recovery period for substantial investment to provide the necessary resources for adequate surgical service provision. We estimate a shortfall of 980 operations in 2020 and 2021, however this may well be an underestimate, as operation numbers were increasing annually by 13.5% per year prior to the pandemic. If the 2017-2019 average of 1837 annual operations was restored, and surgical capacity was increased by 10%, it would take more than 5 years to make up for this shortfall.

Despite national prioritisation guidance stating that surgery for pain associated with severe endometriosis should be performed within 3 months (FSSA, 2020; RCOG, 2021a), it is likely that patients will experience significantly longer wating times than this, as they did prior to the pandemic. Indeed, NHS England statistics from February 2022 show only 59.9% of patients received treatment within 18 weeks of referral for gynaecological disease, down from 64.8% in April 2021 (NHS England, 2021b). We found considerable variation in reductions in operations between different regions of the UK, with the worst affected regions being Wales and the Northwest of England. This is particularly alarming as these regions already had some of the longest waiting lists for gynaecological surgery prior to the pandemic, indicating a particular need for geographically targeted support for services (RCOG, 2022b).

Most endometriosis surgery is performed laparoscopically, and the magnified decline in procedures for complex endometriosis may have been in part due to concerns raised early in the pandemic about aerosol generation during laparoscopy. However, in this study, we did not observe a decline in the laparoscopic approach for the treatment of severe endometriosis during the pandemic. The increased use of pre-operative testing of patients and measures to improve the COVID-safety of laparoscopy have allowed the careful re-introduction of endometriosis surgery at some centres through 2020 and 2021, mitigating some of the disruption (Odejinmi et al., 2020; Saridogan and Grimbizis, 2020). Despite these changes, our study shows that the proportions of patients undergoing the different types of surgery on the bowel (shaving, disc resection and segmental resection) remains unaffected.

The reduction in operations for severe endometriosis is unsurprising given the lack of operating theatre capacity and availability of health service staff with appropriate expertise during the COVID-19 pandemic. However, the lack of patient access to primary care services may be equally important, as well as other obstacles to obtaining a referral for endometriosis treatment, such as the need to self-isolate and reductions in endometriosis clinic capacity. In support of this, a report into elective care in England found that while there was a large decrease in the number of referred patients receiving definitive care in 2020 compared to 2019, there was an even larger decrease in the number of new referrals made, so that the total number of patients waiting for surgery actually decreased. The growing number of patients unable to access treatment for pain is likely to have serious consequences, not only for individuals’ mental health and disease progression, but also for society due to increased strain on community and emergency medical services and lost economic activity due to absence from work, estimated at €6298 per patient per year (Simoens et al., 2012).

As more patients have been able to access GPs and obtain referrals to specialist surgical services, the surge in demand has exceeded capacity, resulting in rapidly growing waiting times and increased rejection of referrals by specialist clinics (BMA, 2022). Several ways of addressing these concerns have been put forward, including clinical prioritisation, re-assessment of patients on waiting lists to ensure they still require surgery, pooling of waiting lists and engagement with the independent sector (Carr et al., 2021). The use of “Covid- light sites” within and across hospitals has been a key development to improving and maintaining access to surgical care (RCSE, 2021), and the combining centralisation, clinical prioritisation and COVID-light sites has been used with success in orthopaedics, creating a possible model for other specialist services (Ahmed et al., 2021). Focussed weekend operating lists have also been put forward as a solution to reduce the backlog, and this strategy has been cost-effective for tackling waiting lists for laparoscopic cholecystectomy (Clifford et al., 2022).

Our study has shown that the number of endometriosis operations per centre decreased by a median 56.1%, while the number of centres remained approximately the same. Only 5 centres had increased operation numbers, which is likely to have been due to newer centres in the process of growing their workload as usually occurs when centres acquire additional staff. Considering the almost universal decline in centre activity, the BSGE decided not to use operation numbers from 2020 or 2021 as part of the accreditation process. This will allow many centres to remain on the BSGE centres list, and thereby ensure that the infrastructure is in place for an increase in operation numbers in the pandemic recovery period. However, part of the UK government’s plan to deliver 30% more elective care by 2024 involves centralisation of surgical services into “hubs” to increase efficiency (NHS England and NHS Improvement, 2022), the future of endometriosis care may involve fewer but more specialised endometriosis centres.

The sharp reduction in surgical caseload for highly complex surgery will undoubtedly have caused ripple-effects on proficiency, training, and hands-on experience for both experienced and trainee-level endometriosis surgeons. Dual surgeon “Buddy” operating, where experienced surgeons operate together, has been suggested by some to help reattain pre-pandemic levels of proficiency and confidence (Ellis et al., 2021). This is a difficult debate as it represents a further cost pressure to organisations to fund what is regarded by some as a necessity. There is clear evidence that the pandemic has had severe consequences for surgical training, with one study finding that more than a quarter of surgical trainees entered their final year of training behind their expected trajectory in 2021 (Clements et al., 2021). New endometriosis surgeons will have felt the impact on their training acutely, with significant delays to the acquisition of new skills. Additional resources may therefore be required for advanced laparoscopic surgery training to maintain any benefit in additional surgical capacity for the longer term.

This study finds that reduction in endometriosis operations at a regional level are correlated with regional COVID-19 case rates. This is likely to be due to several factors, including the repurposing of a higher proportion of operating theatres for the care of critically unwell COVID patients in regions with higher case numbers, as well as greater staff absence due to sickness and caring for unwell household members. Also, regions with worse COVID outbreaks may have seen fewer patients accessing GP services and endometriosis clinics due to a combination of reduced primary care services and patient avoidance of healthcare facilities. Patients undergoing elective surgery who contract COVID have a high mortality risk (Abbott et al., 2021), and concerns over this may have prompted more patients to delay their surgery in regions hit worse by the pandemic. Evidence that regions worse affected by the pandemic suffered greater reductions in endometriosis operations may be helpful in planning the allocation of resources so that operating capacity is increased where it is most needed. Indeed, greater sharing of patient pathways and working beyond single hospital or regional boundaries may be required to smooth out some of the inequities in waiting times and access to care.

In conclusion, the effect of the COVID-19 pandemic on endometriosis surgery has been dramatic and should be addressed with urgency. A co-ordinated effort is required to establish increased capacity, wider access to care and greater support for patients and their clinical teams. This study is important because it documents the scale of the problem, and it is hoped that this will encourage efforts to meet the challenge of promoting awareness and excellence in care for patients with endometriosis.
